# 1,2-Bis(diphenyl­phosphino)-1,2-diethyl­hydrazine

**DOI:** 10.1107/S1600536810015886

**Published:** 2010-05-08

**Authors:** Frederik H. Kriel, Manuel A. Fernandes, Judy Caddy

**Affiliations:** a Project AuTEK, Mintek, Private Bag X3015, Randburg 2125, South Africa; b Molecular Science Institute, School of Chemistry, University of the Witwatersrand, PO Wits, 2050 Johannesburg, South Africa

## Abstract

The title compound, C_28_H_30_N_2_P_2_, adopts a well documented and studied *gauche* conformation around the hydrazine bond.  Bond lengths and angles are in the typical ranges expected for P—N and P—C bonds. A normal hydrazine N—N bond length of 1.426 (3) Å is observed.

## Related literature

For related structures, see: Reddy *et al.* (1994 ▶, 1995 ▶); Pelizzi & Pelizzi (1979 ▶). For *ab initio* mol­ecular modelling studies, see: Cowley *et al.* (1979 ▶).
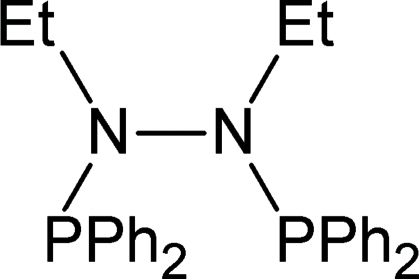


## Experimental

### 

#### Crystal data

C_28_H_30_N_2_P_2_
*M* *_r_* = 456.48
Monoclinic, 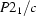

*a* = 14.623 (5) Å
*b* = 13.085 (4) Å
*c* = 13.494 (4) Å
β = 108.182 (6)°
*V* = 2453.1 (13) Å^3^
*Z* = 4
Mo *K*α radiation
μ = 0.20 mm^−1^
*T* = 173 K
0.44 × 0.17 × 0.17 mm

#### Data collection

Bruker SMART 1K CCD area-detector diffractometer
15744 measured reflections
6008 independent reflections
3774 reflections with *I* > 2σ(*I*)
*R*_int_ = 0.056

#### Refinement

*R*[*F* ^2^ > 2σ(*F* ^2^)] = 0.058
*wR*(*F* ^2^) = 0.156
*S* = 1.02
6008 reflections
289 parameters
H-atom parameters constrained
Δρ_max_ = 0.82 e Å^−3^
Δρ_min_ = −0.56 e Å^−3^

### 

Data collection: *SMART-NT* (Bruker, 1998 ▶); cell refinement: *SAINT-Plus* (Bruker, 1999 ▶); data reduction: *SAINT-Plus*; program(s) used to solve structure: *SHELXS97* (Sheldrick, 2008 ▶); program(s) used to refine structure: *SHELXL97* (Sheldrick, 2008 ▶); molecular graphics: *ORTEP-3* (Farrugia, 1997 ▶) and *Mercury* (Macrae *et al.*, 2008 ▶); software used to prepare material for publication: *WinGX* (Farrugia, 1999 ▶).

## Supplementary Material

Crystal structure: contains datablocks I, global. DOI: 10.1107/S1600536810015886/wn2384sup1.cif

Structure factors: contains datablocks I. DOI: 10.1107/S1600536810015886/wn2384Isup2.hkl

Additional supplementary materials:  crystallographic information; 3D view; checkCIF report

## Figures and Tables

**Fig. 1.**
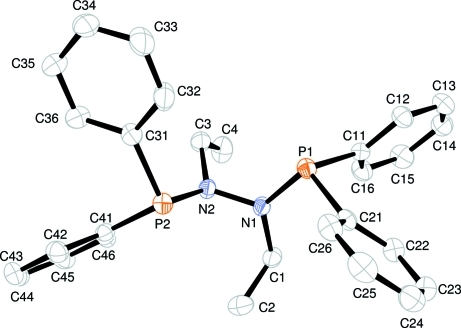
Molecular structure of the title compound, drawn with displacement ellipsoids at the 50% probability level. Hydrogen atoms have been omitted for clarity.

## supplementary crystallographic information

### Comment

Crystals of the title compound (Fig. 1) are found to be monoclinic, crystallising in the space group *P*2_1_*/c*. Crystals of 1,2-bis(diphenylphosphino)ethane (dppe) show similar characteristics; it is monoclinic and crystallises in the spacegroup *P*2_1_*/n *(Pelizzi *et al.*, 1979). The title compound has four molecules per unit cell compared to two in dppe; the latter has a centre of symmetry at the mid-point of the C(sp^3^)—C(sp^3^) bond.

Dppe was shown to adopt a staggered conformation, whereas the title compound has a *gauche* conformation. This *gauche* conformation adopted by hydrazine has been well documented and studied both experimentally and by *ab anitio* molecular modelling (Cowley *et al.*, 1979). It was found that the ground-state geometry of hydrazine is *gauche*, with a dihedral angle close to 90°. *Ab initio* theoretical estimates of the *gauche-anti* and *gauche-syn* barrier heights fall in the ranges 1.6-6.2 and 9.7-13.7 kcal/mol, respectively. In hydrazine, the relative stability of the conformations are *gauche *> *anti* > *syn *(Cowley *et al.*, 1979). The planar conformation of dppe allows it to form stacks of molecules; this is not possible for the title compound.

Bond lengths and angles are in the typical ranges expected for P—N and P—C bonds (Reddy *et al.*, 1994). A normal hydrazine N—N bond length of 1.426 (3) Å is observed.

### Experimental

The title compound was synthesised in a similar manner to published methods (Reddy *et al.* 1994, 1995). The compound was obtained as light yellow, single crystalline flakes from the worked-up diethylether layer. The diethylether layer was concentrated and kept at -20 °C for 1-3 days. The supernatant was removed from the crystalline flakes and placed back in the freezer for further crystallisation. 86% yield. Mp 95-96 °C.

### Refinement

The H atoms were positioned geometrically and allowed to ride on their respective parent atoms, with C—H = 0.93 (Ar-H) or 0.96 (CH_3_) Å, and with U_eq_ = 1.2 (Ar-H) or 1.5 (CH_3_)U_eq_(C).

### Crystal data

**Table:**

C_28_H_30_N_2_P_2_	*Z* = 4
*M**_r_* = 456.48	*F*(000) = 968
Monoclinic, *P*2_1_/*c*	*D*_x_ = 1.236 Mg m^−^^3^
Hall symbol: -P 2ybc	Melting point: 368 K
*a* = 14.623 (5) Å	Mo *K*α radiation, λ = 0.71073 Å
*b* = 13.085 (4) Å	µ = 0.20 mm^−^^1^
*c* = 13.494 (4) Å	*T* = 173 K
β = 108.182 (6)°	Prismic, colourless
*V* = 2453.1 (13) Å^3^	0.44 × 0.17 × 0.17 mm

### Data collection

**Table:**

Bruker SMART 1K CCD area-detector diffractometer	3774 reflections with *I* > 2σ(*I*)
Radiation source: fine-focus sealed tube	*R*_int_ = 0.056
graphite	θ_max_ = 28.3°, θ_min_ = 1.5°
phi and ω scans	*h* = −17→19
15744 measured reflections	*k* = −13→17
6008 independent reflections	*l* = −17→14

### Refinement

**Table:**

Refinement on *F*^2^	Primary atom site location: structure-invariant direct methods
Least-squares matrix: full	Secondary atom site location: difference Fourier map
*R*[*F*^2^ > 2σ(*F*^2^)] = 0.058	Hydrogen site location: inferred from neighbouring sites
*wR*(*F*^2^) = 0.156	H-atom parameters constrained
*S* = 1.02	*w* = 1/[σ^2^(*F*_o_^2^) + (0.0731*P*)^2^ + 1.0514*P*] where *P* = (*F*_o_^2^ + 2*F*_c_^2^)/3
6008 reflections	(Δ/σ)_max_ = 0.001
289 parameters	Δρ_max_ = 0.82 e Å^−^^3^
0 restraints	Δρ_min_ = −0.56 e Å^−^^3^

### Special details

**Table:**

Experimental. ^1^HNMR (CDCl_3_, 300 MHz) δ_H_ 7.54 (bs, Arom, 4H) 7.36 (bs, Arom, 4H), 7.26 (m, Arom,**12H), 3.73 and 3.23(m, CH_2_CH_3_,4*H*), 0.79 (t, CH_2_CH_3_,^3^*J* (^1^H-^1^H) = 7.0 Hz, 6H). ^13^C NMR (CDCl_3_, 75 MHz) δ_C_ 140.2 (m, Arom), 133.4 (m, Arom), 131.4 (s, Arom), 128.7(s, Arom), 48.7 (t, CH_2_CH_3_, ^2^*J* (^13^C-^31^P) = 2.5 Hz), 14.3 (d, CH_2_CH_3_, ^3^*J* (^13^C-^31^P) = 4.1 Hz). ^31^P NMR (CDCl_3_,162 MHz) δ_P_ 63.4. MS 427 (9%, M – 1). Intensity data were collected on a Bruker SMART1K CCD area detector diffractometer with graphite monochromated Mo *K*α radiation (40kV, 40mA). The collection method involved ω-scans of width 0.3°.
Geometry. All esds (except the esd in the dihedral angle between two l.s. planes) are estimated using the full covariance matrix. The cell esds are taken into account individually in the estimation of esds in distances, angles and torsion angles; correlations between esds in cell parameters are only used when they are defined by crystal symmetry. An approximate (isotropic) treatment of cell esds is used for estimating esds involving l.s. planes.
Refinement. Refinement of *F*^2^ against ALL reflections. The weighted *R*-factor wR and goodness of fit *S* are based on *F*^2^, conventional *R*-factors *R* are based on *F*, with *F* set to zero for negative *F*^2^. The threshold expression of *F*^2^ > σ(*F*^2^) is used only for calculating *R*-factors(gt) etc. and is not relevant to the choice of reflections for refinement. *R*-factors based on *F*^2^ are statistically about twice as large as those based on *F*, and *R*- factors based on ALL data will be even larger.

### Fractional atomic coordinates and isotropic or equivalent isotropic displacement parameters (Å^2^)

**Table:**

	*x*	*y*	*z*	*U*_iso_*/*U*_eq_	
C1	0.86723 (18)	0.1568 (2)	0.1440 (2)	0.0281 (6)	
H1A	0.9183	0.2055	0.1745	0.034*	
H1B	0.8375	0.1759	0.0716	0.034*	
C2	0.9114 (2)	0.0518 (2)	0.1476 (3)	0.0393 (7)	
H2A	0.9577	0.0529	0.1105	0.059*	
H2B	0.8618	0.0032	0.1157	0.059*	
H2C	0.9426	0.0328	0.2189	0.059*	
C3	0.62250 (18)	0.1538 (2)	0.1085 (2)	0.0281 (6)	
H3A	0.6062	0.1945	0.1607	0.034*	
H3B	0.5718	0.1037	0.0821	0.034*	
C4	0.6258 (2)	0.2228 (2)	0.0199 (2)	0.0398 (7)	
H4A	0.5646	0.2558	−0.0088	0.060*	
H4B	0.6399	0.1829	−0.0332	0.060*	
H4C	0.6749	0.2735	0.0456	0.060*	
C11	0.81426 (17)	0.36831 (18)	0.27049 (19)	0.0227 (5)	
C12	0.81650 (19)	0.44196 (19)	0.3461 (2)	0.0288 (6)	
H12	0.8109	0.4215	0.4099	0.035*	
C13	0.8268 (2)	0.5447 (2)	0.3280 (2)	0.0351 (7)	
H13	0.8286	0.5924	0.3796	0.042*	
C14	0.8345 (2)	0.5768 (2)	0.2334 (2)	0.0351 (7)	
H14	0.8425	0.6457	0.2215	0.042*	
C15	0.8303 (2)	0.5057 (2)	0.1571 (2)	0.0364 (7)	
H15	0.8344	0.5272	0.0929	0.044*	
C16	0.8199 (2)	0.4023 (2)	0.1743 (2)	0.0309 (6)	
H16	0.8168	0.3554	0.1216	0.037*	
C21	0.92822 (18)	0.21580 (18)	0.39268 (19)	0.0236 (5)	
C22	1.00751 (19)	0.2724 (2)	0.3876 (2)	0.0279 (6)	
H22	0.9995	0.3222	0.3363	0.034*	
C23	1.0980 (2)	0.2556 (2)	0.4579 (2)	0.0320 (6)	
H23	1.1505	0.2926	0.4525	0.038*	
C24	1.1104 (2)	0.1838 (2)	0.5361 (2)	0.0358 (7)	
H24	1.1711	0.1735	0.5840	0.043*	
C25	1.0331 (2)	0.1275 (2)	0.5431 (2)	0.0386 (7)	
H25	1.0416	0.0792	0.5958	0.046*	
C26	0.9424 (2)	0.1428 (2)	0.4716 (2)	0.0314 (6)	
H26	0.8906	0.1041	0.4764	0.038*	
C31	0.60617 (19)	−0.01800 (19)	0.2575 (2)	0.0257 (5)	
C32	0.6057 (2)	0.0375 (2)	0.3455 (2)	0.0409 (8)	
H32	0.6591	0.0766	0.3805	0.049*	
C33	0.5265 (3)	0.0351 (2)	0.3814 (3)	0.0503 (9)	
H33	0.5271	0.0735	0.4396	0.060*	
C34	0.4471 (2)	−0.0231 (2)	0.3320 (2)	0.0401 (7)	
H34	0.3944	−0.0245	0.3567	0.048*	
C35	0.44666 (19)	−0.0794 (2)	0.2454 (2)	0.0334 (6)	
H35	0.3937	−0.1199	0.2119	0.040*	
C36	0.52481 (18)	−0.0760 (2)	0.2081 (2)	0.0299 (6)	
H36	0.5229	−0.1133	0.1488	0.036*	
C41	0.68240 (17)	−0.10439 (18)	0.1100 (2)	0.0233 (5)	
C42	0.67952 (19)	−0.20902 (19)	0.1322 (2)	0.0299 (6)	
H42	0.6948	−0.2303	0.2012	0.036*	
C43	0.6543 (2)	−0.2810 (2)	0.0532 (2)	0.0353 (7)	
H43	0.6526	−0.3499	0.0695	0.042*	
C44	0.63169 (19)	−0.2509 (2)	−0.0500 (2)	0.0359 (7)	
H44	0.6137	−0.2992	−0.1031	0.043*	
C45	0.6360 (2)	−0.1489 (2)	−0.0735 (2)	0.0330 (6)	
H45	0.6217	−0.1285	−0.1427	0.040*	
C46	0.66139 (18)	−0.07618 (19)	0.0055 (2)	0.0271 (6)	
H46	0.6644	−0.0077	−0.0115	0.033*	
N1	0.79504 (14)	0.16499 (15)	0.19838 (16)	0.0237 (5)	
N2	0.71357 (14)	0.09995 (15)	0.15842 (16)	0.0229 (5)	
P1	0.80362 (5)	0.23470 (5)	0.30655 (5)	0.02321 (17)	
P2	0.71714 (5)	−0.01492 (5)	0.21993 (5)	0.02383 (17)	

### Atomic displacement parameters (Å^2^)

**Table:**

	*U*^11^	*U*^22^	*U*^33^	*U*^12^	*U*^13^	*U*^23^
C1	0.0266 (13)	0.0350 (15)	0.0259 (14)	−0.0074 (11)	0.0127 (11)	−0.0080 (11)
C2	0.0296 (15)	0.0451 (18)	0.0467 (19)	0.0033 (13)	0.0168 (14)	−0.0038 (14)
C3	0.0241 (13)	0.0261 (13)	0.0317 (15)	0.0017 (10)	0.0052 (11)	0.0013 (11)
C4	0.0381 (17)	0.0316 (16)	0.0414 (18)	−0.0010 (13)	0.0001 (14)	0.0093 (13)
C11	0.0218 (12)	0.0226 (12)	0.0240 (13)	0.0010 (10)	0.0075 (10)	−0.0005 (10)
C12	0.0335 (15)	0.0278 (14)	0.0261 (14)	0.0001 (11)	0.0110 (12)	−0.0017 (11)
C13	0.0374 (16)	0.0284 (15)	0.0401 (18)	0.0025 (12)	0.0130 (13)	−0.0074 (12)
C14	0.0343 (16)	0.0218 (14)	0.0486 (19)	0.0028 (11)	0.0123 (14)	0.0042 (13)
C15	0.0464 (17)	0.0309 (15)	0.0353 (16)	0.0004 (12)	0.0179 (14)	0.0079 (12)
C16	0.0396 (16)	0.0296 (14)	0.0260 (14)	−0.0013 (12)	0.0140 (12)	−0.0016 (11)
C21	0.0308 (14)	0.0213 (13)	0.0196 (13)	0.0007 (10)	0.0092 (11)	−0.0026 (10)
C22	0.0324 (14)	0.0265 (13)	0.0245 (14)	−0.0024 (11)	0.0083 (11)	0.0003 (11)
C23	0.0305 (14)	0.0307 (15)	0.0334 (15)	−0.0024 (11)	0.0081 (12)	−0.0041 (12)
C24	0.0369 (16)	0.0379 (16)	0.0258 (15)	0.0073 (13)	−0.0001 (12)	−0.0039 (12)
C25	0.0533 (19)	0.0341 (16)	0.0253 (15)	0.0074 (14)	0.0079 (13)	0.0046 (12)
C26	0.0436 (16)	0.0287 (14)	0.0242 (14)	−0.0019 (12)	0.0139 (12)	0.0010 (11)
C31	0.0328 (14)	0.0246 (13)	0.0224 (13)	−0.0014 (11)	0.0125 (11)	0.0026 (10)
C32	0.0508 (19)	0.0384 (17)	0.0418 (18)	−0.0200 (14)	0.0265 (15)	−0.0142 (14)
C33	0.071 (2)	0.0453 (19)	0.051 (2)	−0.0190 (17)	0.0426 (19)	−0.0208 (16)
C34	0.0452 (18)	0.0382 (17)	0.0482 (19)	−0.0025 (14)	0.0308 (15)	−0.0008 (14)
C35	0.0261 (14)	0.0415 (16)	0.0332 (16)	−0.0012 (12)	0.0101 (12)	0.0012 (13)
C36	0.0273 (14)	0.0402 (16)	0.0211 (14)	0.0002 (11)	0.0062 (11)	−0.0015 (11)
C41	0.0207 (12)	0.0237 (13)	0.0279 (14)	0.0004 (10)	0.0111 (10)	0.0016 (11)
C42	0.0325 (15)	0.0263 (14)	0.0331 (16)	0.0024 (11)	0.0136 (12)	0.0036 (11)
C43	0.0344 (15)	0.0205 (14)	0.0523 (19)	−0.0018 (11)	0.0153 (14)	−0.0029 (13)
C44	0.0286 (15)	0.0364 (16)	0.0409 (17)	−0.0016 (12)	0.0080 (13)	−0.0150 (13)
C45	0.0352 (15)	0.0358 (16)	0.0281 (15)	0.0008 (12)	0.0098 (12)	−0.0065 (12)
C46	0.0305 (14)	0.0223 (13)	0.0304 (15)	0.0002 (10)	0.0122 (12)	−0.0014 (11)
N1	0.0240 (11)	0.0265 (11)	0.0232 (11)	−0.0061 (9)	0.0110 (9)	−0.0069 (9)
N2	0.0212 (10)	0.0203 (10)	0.0256 (11)	−0.0024 (8)	0.0051 (9)	0.0004 (9)
P1	0.0277 (3)	0.0231 (3)	0.0212 (3)	−0.0023 (3)	0.0112 (3)	−0.0013 (3)
P2	0.0256 (3)	0.0242 (3)	0.0214 (3)	−0.0009 (3)	0.0069 (3)	0.0011 (3)

### Geometric parameters (Å, °)

**Table:**

C1—N1	1.465 (3)	C24—C25	1.378 (4)
C1—C2	1.512 (4)	C24—H24	0.9300
C1—H1A	0.9700	C25—C26	1.390 (4)
C1—H1B	0.9700	C25—H25	0.9300
C2—H2A	0.9600	C26—H26	0.9300
C2—H2B	0.9600	C31—C32	1.393 (4)
C2—H2C	0.9600	C31—C36	1.393 (4)
C3—N2	1.471 (3)	C31—P2	1.846 (3)
C3—C4	1.510 (4)	C32—C33	1.390 (4)
C3—H3A	0.9700	C32—H32	0.9300
C3—H3B	0.9700	C33—C34	1.375 (4)
C4—H4A	0.9600	C33—H33	0.9300
C4—H4B	0.9600	C34—C35	1.381 (4)
C4—H4C	0.9600	C34—H34	0.9300
C11—C12	1.396 (3)	C35—C36	1.386 (4)
C11—C16	1.398 (4)	C35—H35	0.9300
C11—P1	1.834 (3)	C36—H36	0.9300
C12—C13	1.383 (4)	C41—C46	1.395 (4)
C12—H12	0.9300	C41—C42	1.405 (3)
C13—C14	1.380 (4)	C41—P2	1.833 (3)
C13—H13	0.9300	C42—C43	1.383 (4)
C14—C15	1.374 (4)	C42—H42	0.9300
C14—H14	0.9300	C43—C44	1.385 (4)
C15—C16	1.390 (4)	C43—H43	0.9300
C15—H15	0.9300	C44—C45	1.378 (4)
C16—H16	0.9300	C44—H44	0.9300
C21—C22	1.396 (4)	C45—C46	1.391 (4)
C21—C26	1.397 (4)	C45—H45	0.9300
C21—P1	1.847 (3)	C46—H46	0.9300
C22—C23	1.384 (4)	N1—N2	1.426 (3)
C22—H22	0.9300	N1—P1	1.692 (2)
C23—C24	1.381 (4)	N2—P2	1.710 (2)
C23—H23	0.9300		
			
N1—C1—C2	114.7 (2)	C24—C25—C26	120.0 (3)
N1—C1—H1A	108.6	C24—C25—H25	120.0
C2—C1—H1A	108.6	C26—C25—H25	120.0
N1—C1—H1B	108.6	C25—C26—C21	120.7 (3)
C2—C1—H1B	108.6	C25—C26—H26	119.6
H1A—C1—H1B	107.6	C21—C26—H26	119.6
C1—C2—H2A	109.5	C32—C31—C36	117.5 (2)
C1—C2—H2B	109.5	C32—C31—P2	117.4 (2)
H2A—C2—H2B	109.5	C36—C31—P2	124.9 (2)
C1—C2—H2C	109.5	C33—C32—C31	120.8 (3)
H2A—C2—H2C	109.5	C33—C32—H32	119.6
H2B—C2—H2C	109.5	C31—C32—H32	119.6
N2—C3—C4	113.6 (2)	C34—C33—C32	120.9 (3)
N2—C3—H3A	108.8	C34—C33—H33	119.5
C4—C3—H3A	108.8	C32—C33—H33	119.5
N2—C3—H3B	108.8	C33—C34—C35	119.1 (3)
C4—C3—H3B	108.8	C33—C34—H34	120.5
H3A—C3—H3B	107.7	C35—C34—H34	120.5
C3—C4—H4A	109.5	C34—C35—C36	120.3 (3)
C3—C4—H4B	109.5	C34—C35—H35	119.9
H4A—C4—H4B	109.5	C36—C35—H35	119.9
C3—C4—H4C	109.5	C35—C36—C31	121.4 (3)
H4A—C4—H4C	109.5	C35—C36—H36	119.3
H4B—C4—H4C	109.5	C31—C36—H36	119.3
C12—C11—C16	117.6 (2)	C46—C41—C42	117.5 (2)
C12—C11—P1	116.66 (19)	C46—C41—P2	124.53 (19)
C16—C11—P1	125.7 (2)	C42—C41—P2	118.0 (2)
C13—C12—C11	121.4 (3)	C43—C42—C41	121.2 (3)
C13—C12—H12	119.3	C43—C42—H42	119.4
C11—C12—H12	119.3	C41—C42—H42	119.4
C14—C13—C12	120.2 (3)	C42—C43—C44	120.2 (3)
C14—C13—H13	119.9	C42—C43—H43	119.9
C12—C13—H13	119.9	C44—C43—H43	119.9
C15—C14—C13	119.3 (3)	C45—C44—C43	119.6 (3)
C15—C14—H14	120.3	C45—C44—H44	120.2
C13—C14—H14	120.3	C43—C44—H44	120.2
C14—C15—C16	121.0 (3)	C44—C45—C46	120.4 (3)
C14—C15—H15	119.5	C44—C45—H45	119.8
C16—C15—H15	119.5	C46—C45—H45	119.8
C15—C16—C11	120.4 (3)	C45—C46—C41	121.0 (2)
C15—C16—H16	119.8	C45—C46—H46	119.5
C11—C16—H16	119.8	C41—C46—H46	119.5
C22—C21—C26	118.2 (2)	N2—N1—C1	114.50 (19)
C22—C21—P1	124.72 (19)	N2—N1—P1	118.43 (15)
C26—C21—P1	117.0 (2)	C1—N1—P1	126.87 (16)
C23—C22—C21	120.8 (2)	N1—N2—C3	114.66 (19)
C23—C22—H22	119.6	N1—N2—P2	116.47 (15)
C21—C22—H22	119.6	C3—N2—P2	121.99 (16)
C24—C23—C22	120.1 (3)	N1—P1—C11	105.93 (11)
C24—C23—H23	119.9	N1—P1—C21	105.29 (11)
C22—C23—H23	119.9	C11—P1—C21	98.39 (11)
C25—C24—C23	120.1 (3)	N2—P2—C41	102.11 (11)
C25—C24—H24	120.0	N2—P2—C31	104.86 (11)
C23—C24—H24	120.0	C41—P2—C31	99.40 (11)
			
C16—C11—C12—C13	−1.9 (4)	C2—C1—N1—N2	−60.7 (3)
P1—C11—C12—C13	178.3 (2)	C2—C1—N1—P1	114.0 (2)
C11—C12—C13—C14	0.4 (4)	C1—N1—N2—C3	−113.0 (2)
C12—C13—C14—C15	1.0 (4)	P1—N1—N2—C3	71.8 (2)
C13—C14—C15—C16	−1.0 (4)	C1—N1—N2—P2	95.4 (2)
C14—C15—C16—C11	−0.4 (4)	P1—N1—N2—P2	−79.8 (2)
C12—C11—C16—C15	1.8 (4)	C4—C3—N2—N1	57.0 (3)
P1—C11—C16—C15	−178.4 (2)	C4—C3—N2—P2	−153.17 (19)
C26—C21—C22—C23	1.0 (4)	N2—N1—P1—C11	−123.59 (17)
P1—C21—C22—C23	177.6 (2)	C1—N1—P1—C11	61.9 (2)
C21—C22—C23—C24	−1.7 (4)	N2—N1—P1—C21	132.80 (17)
C22—C23—C24—C25	1.2 (4)	C1—N1—P1—C21	−41.7 (2)
C23—C24—C25—C26	0.0 (4)	C12—C11—P1—N1	176.22 (19)
C24—C25—C26—C21	−0.6 (4)	C16—C11—P1—N1	−3.6 (3)
C22—C21—C26—C25	0.1 (4)	C12—C11—P1—C21	−75.2 (2)
P1—C21—C26—C25	−176.7 (2)	C16—C11—P1—C21	105.1 (2)
C36—C31—C32—C33	−0.5 (4)	C22—C21—P1—N1	85.1 (2)
P2—C31—C32—C33	−177.0 (3)	C26—C21—P1—N1	−98.2 (2)
C31—C32—C33—C34	0.9 (5)	C22—C21—P1—C11	−24.0 (2)
C32—C33—C34—C35	−0.3 (5)	C26—C21—P1—C11	152.6 (2)
C33—C34—C35—C36	−0.9 (5)	N1—N2—P2—C41	−131.64 (17)
C34—C35—C36—C31	1.4 (4)	C3—N2—P2—C41	79.0 (2)
C32—C31—C36—C35	−0.7 (4)	N1—N2—P2—C31	125.09 (17)
P2—C31—C36—C35	175.6 (2)	C3—N2—P2—C31	−24.3 (2)
C46—C41—C42—C43	−1.7 (4)	C46—C41—P2—N2	0.8 (2)
P2—C41—C42—C43	−179.7 (2)	C42—C41—P2—N2	178.72 (19)
C41—C42—C43—C44	0.2 (4)	C46—C41—P2—C31	108.3 (2)
C42—C43—C44—C45	1.1 (4)	C42—C41—P2—C31	−73.7 (2)
C43—C44—C45—C46	−1.0 (4)	C32—C31—P2—N2	−78.5 (2)
C44—C45—C46—C41	−0.5 (4)	C36—C31—P2—N2	105.2 (2)
C42—C41—C46—C45	1.8 (4)	C32—C31—P2—C41	176.2 (2)
P2—C41—C46—C45	179.7 (2)	C36—C31—P2—C41	−0.1 (3)
